# CADASIL or *NOTCH3* mutaion spectrum diseases? Interpretation of *NOTCH3* mutations and clinical heterogeneity in CADASIL

**DOI:** 10.3389/fneur.2025.1662012

**Published:** 2025-09-12

**Authors:** Yuehui Wang, Yiran Liu, Hongbin Mo, Yue Han, Yuanyuan Jing, Fang Deng

**Affiliations:** Department of Neurology, The First Hospital of Jilin University, Changchun, China

**Keywords:** CADASIL, *NOTCH3*, *NOTCH3* mutaion spectrum diseases, hereditary cerebral small vessel disease, the phenotypic heterogeneity

## Abstract

Cerebral autosomal dominant arteriopathy with subcortical infarcts and leukoencephalopathy (CADASIL) is an autosomal dominant disorder characterized by midlife-onset cerebrovascular disease and dementia. It is caused by mutations in the *NOTCH3* gene, which affects the amount of cysteine in the extracellular domain (ECD) of the receptor, leading to protein misfolding and receptor aggregation. Emerging evidence indicates that beyond classical missense mutations, other variants including cysteine-sparing missense mutations, homozygous mutations, small deletions, duplications, splice site mutations, a deletion/insertion and loss-of-function mutations may lead to distinct phenotypes with variable severity and disease penetrance. The marked heterogeneity in genotypes and phenotypes poses significant challenges for CADASIL diagnosis and clinical management. The aim of this review is to summarize the mutational spectrum of CADASIL, explore the possible genotype–phenotype correlations and discuss the phenotypic heterogeneity of *NOTCH3* mutations. More studies are needed in the future to demonstrate whether CADASIL can be expanded from classical cerebral small vessel disease to a new spectrum of diseases that share the same pathogenesis as mutations in the NOTCH3 gene.

## 1 Introduction

Cerebral autosomal dominant arteriopathy with subcortical infarcts and leukoencephalopathy (CADASIL) is a monogenic inherited small vessel disease, now recognized as the most prevalent genetic cause of stroke and dementia in adults. This autosomal dominant disorder is caused by mutations in the *NOTCH3* gene (1). To date, over 220 mutations have been reported in the *NOTCH3* gene, of which *NOTCH3* missense mutations altering cysteine residue count serve as the primary diagnostic criterion and most common mutation type (2). The accumulation of granular osmiophilic material (GOM), which contains the *NOTCH3* extracellular domain (*NOTCH3*ECD), is detected via skin biopsies and is considered a hallmark pathological feature of CADASIL. Notably, GOM primarily consists of the extracellular structural domain (ECD) of *NOTCH3* (3). However, mutations that preserve cysteine and other mutations including homozygous mutations, small deletions, duplications, splice site mutations, a deletion/insertion and loss-of-function mutations in *NOTCH3* may also induce structural alterations in *NOTCH3*, leading to clinical manifestations resembling those of CADASIL. Thus, these diverse genotypes have challenged conventional diagnostic criteria for CADASIL-associated mutations.

CADASIL is a cerebral small-vessel disease in which patients may present with multiple clinical features, including migraine with aura, cerebrovascular events in young to middle-aged individuals, mood disorders, affective apathy, cognitive decline progressing to dementia, and diffuse white matter lesions with subcortical infarcts on neuroimaging findings (4). Elucidation of genotype–phenotype correlations and their mechanisms is essential to explain the clinical heterogeneity of CADASIL. Recent studies have found various manifestations of intracranial large artery anomalies, coronary artery disease, and abnormalities in lipid and glucose metabolism in patients with mutation of *NOTCH3* gene. This suggests that *NOTCH3* mutations cause more than just classical cerebral small-vessel disease and may involve large intracranial arteries and extracranial diseases. Therefore, further studies are needed to investigate whether the CADASIL phenotype is not limited to the small cerebral vessels. Moreover, the establishment of novel terminology to describe the phenotypic heterogeneity associated with this gene mutation may be necessary.

Therefore, we conducted a comprehensive evaluation of the diversity of mutations in the *NOTCH3* gene, the polymorphisms in the clinical phenotype of CADASIL, and the relationship between genotype and phenotype. We first systematically cataloged *NOTCH3* mutation types to expand the established CADASIL mutational spectrum. Second, we investigated genotype–phenotype correlations through integrated pathogenesis studies, providing mechanistic insights into clinical heterogeneity. Finally, based on the findings of intracranial large artery anomalies, coronary artery disease and metabolic abnormalities caused by *NOTCH3* mutations, we need to revisit the concept of CADASIL disease. Whether this association is part of the *NOTCH3* mutation phenotype or whether it affects the clinical course is uncertain, but should be investigated further.

## 2 Diversity of mutations in the *NOTCH3* gene

### 2.1 Typical NOTCH gene mutations

Classical CADASIL is caused by mutations in the *NOTCH3* gene located on chromosome 19p13, which is involved in the differentiation and maturation of vascular smooth muscle cells, vascular development during embryogenesis, and vascular integrity. The *NOTCH3* gene contains 33 exons and encodes a transmembrane receptor consisting of three structural domains, including the NOTCH extracellular domain (NECD), the transmembrane domain, and the NOTCH intracellular domain (NICD) (5). Among these, exons 2–24 encode *Notch3*^ECD^, which includes 34 epidermal growth factor-like repeat**s** (EGFr), each of which typically includes highly conserved six cysteine residues that form three disulfide bonds. Mutations in the *NOTCH3* gene in CADASIL always cause an increase or deletion in the number of cysteine residues within 1 of the EGFr, resulting in an odd number of cysteine residues (usually 5 or 7). That unpaired cysteine residue, which is expected to disrupt normal disulfide bond formation, leads to misfolding of EGFr and increased *NOTCH3* multimerization, ultimately causing GOM formation (6). GOM formation has been recognized as a typical pathologic features of CADASIL and has been validated in most patients with classical CADASIL.

Heterozygous missense mutations causing altered cysteine numbers have long been recognized as the typical mutational form of CADASIL, and this pathogenic mutation is located on exons 2–24 encoding the 34 EGFR structural domains. In particular, exon 2–6 is considered a mutational hotspot (7).

### 2.2 Cysteine-sparing *NOTCH3* gene mutations

With advances in gene sequencing and more and more studies, we have found that some patients harboring *NOTCH3* missense mutations that preserve cysteine, in particular the p. Arg61Trp, p. Arg75Pro, p. Asp80Gly and p. Arg213Lys mutations, exhibit clinical syndromes consistent with CADASIL, and skin biopsies of these patients have also found GOM deposits in their skin (8). The newly identified p. Gly73Ala, p. Arg75Gln, p. Ser1418Leu and p. Arg1761His mutations are also potentially pathogenic (9). Converging evidence suggests that in the genetic profile of *NOTCH3* cysteine-sparing mutations, mutations are more frequently detected in exon 3, with p. Arg75Pro being the most common type.

### 2.3 Other *NOTCH3* mutation types

In addition to this, many other mutations in *NOTCH3* have been reported in recent years, causing CADASIL-like clinical phenotypes. The first category is some reports on homozygous mutations. There is a case report of a homozygous missense mutation c. C1759T (p. Arg587Cys) in *NOTCH3* gene found in two brothers from China, both patients showed stroke, gait instability, cognitive impairment and psychiatric disorders. Fused white matter hypersignal and multiple lacunar infarcts were demonstrated on MRI, especially by skin biopsy which revealed characteristic GOM deposits in these two patients (10). Consistently, Tuominen et al. also described a patient with a homozygous mutation in *NOTCH3* causing a CADASIL-like clinical phenotype, with the mutation located in c. C475T (p. Arg133Cys), and found GOM deposits (11). However, there have also been case reports of earlier onset and more severe disease phenotypes in patients with pure mutations compared with classic CADASIL (12). Overall, the phenotypes of patients with pure synaptic mutations described to date are almost exclusively within the normal CADASIL spectrum, and the reasons for the variation in clinical severity need to be further explored. The second group consists of small *NOTCH3* deletions, duplications, splice site mutations and a deletion/insertion leading to a numerical cysteine alteration. These patients exhibit a classic CADASIL-like clinical phenotype and most of them underwent biopsy with clear GOM deposition (13, 14). Consistent with classical *NOTCH3* mutations, almost all of these reported mutations involve alterations in the number of cysteine residues in EGFr, and thus they may be lethal. The paucity of reports of such mutations may be due in part to technical limitations of direct sequencing that miss intronic mutations, large deletions, multiplications, or large rearrangements of the *NOTCH3* gene. However, a CADASIL phenotype caused by a 12-base pair deletion that does not result in a change in the number of cysteines was reported in one study. This deletion may alter the spacing between the two cysteines in EGFr, resulting in disruption of the normal disulfide bond (15). The last category is *NOTCH3* loss-of-function mutations, including nonsense mutations causing premature stop codons and frameshift deletion. Such mutations do not trigger changes in the number of EGFr cysteine residues and GOM deposition, but cause hereditary small vessel disease phenotypes of varying severity or penetrance, similar to CADASIL (16–19). Although some *NOTCH3* mutations outside the exons encoding EGFr have also been reported, whether these mutations cause the CADASIL phenotype remains highly controversial, and more patients with such mutations should be found and examined to clarify the conclusions (20–23).

In summary, in the past 30 years since the identification of classical mutations in the CADASIL-causing gene *NOTCH3*, we have identified an increasing number of novel *NOTCH3* variant types that exhibit clinical manifestations similar t1o those of CADASIL, implying a diversification of the *NOTCH3* genotypes that cause CADASIL, partly explaining why this disease is much more prevalent than initially assumed, and is even considered to be the most common type of inherited vascular dementia (Table 1). Therefore, we believe that continuous additions to the CADASIL mutational spectrum are essential to clarify the diagnosis of this disease. However, to avoid erroneous mutation interpretations and diagnoses, our analysis of these patients with *NOTCH3* mutations should always include comprehensive *NOTCH3* molecular screening, as well as comprehensive clinical (re)evaluation, including skin biopsie.

**Table 1:** Classification of *NOTCH3* mutations associated with CADASIL.

*NOTCH3* mutation type		Common mutation locations	Ref.
Typical *NOTCH3* missense mutations		c. C1819T(p. Arg607Cys),c. C544T(p. Arg182Cys),c. C268T(p. Arg90Cys),c. C505T(p. Arg169Cys),c. C457T(p. Arg153Cys),c. C421T(p. Arg141Cys),c. C397T(p. Arg133Cys)	(81, 82)
*NOTCH3* Cysteine-Sparing Mutations		c. C181T(p. Arg61Trp),c. G224C(p. Arg75Pro),c. A239G(p. Asp80Gly),c. G638A(p. Arg213Lys)	(48, 83–85)
Other mutations	*NOTCH3* homozygous mutations	c. C1759T(p. Arg587Cys),c. C475T(p. Arg133Cys),c. C2898A(p. Cys966*),c. C3769T(p. Arg1231Cys),c. G3944A(p. Cys1315Tyr),c. T547A(p. Cys183Ser),c. G1582T(p. Gly528Cys),c. C1732T(p. Arg578Cys),c. C1630T(p. Arg544Cys)	(10–12, 41, 86–90)
	Small *NOTCH3* deletions, duplications, splice site mutations and a deletion/insertion leading to a numerical cysteine alteration	c.226_234del(p. Cys76_Leu78del),c.231_248del(p. Gln77_Cys82del),c.277_279dup(p. Cys93dup),c.239_253del(p. Asp80_Ser84del),c.459_467del(p. Arg153_Cys155del),c.341-2A > G(P. Gly114_Pro120del),c.714_758del(p. Asp239_Asp253del),c.955_956delGCinsTG(p. Ala319Cys),c.1057_1071dup(p. Asp353_Ser357dup),c.1300_1298dup(p. Glu434_Leu436dup),c.2411-1G > T(p. Gly804_Asn856delinsAsp)	(13, 14, 91–97)
	*NOTCH3* loss-of-function mutations(premature stop codons and frameshift deletion)	c.2968delG p. Glu990Argfs^*^282, c. C307T(p. Arg103X)	(16–19)

## 3 Pathogenesis of CADASIL

**Figure 1:**
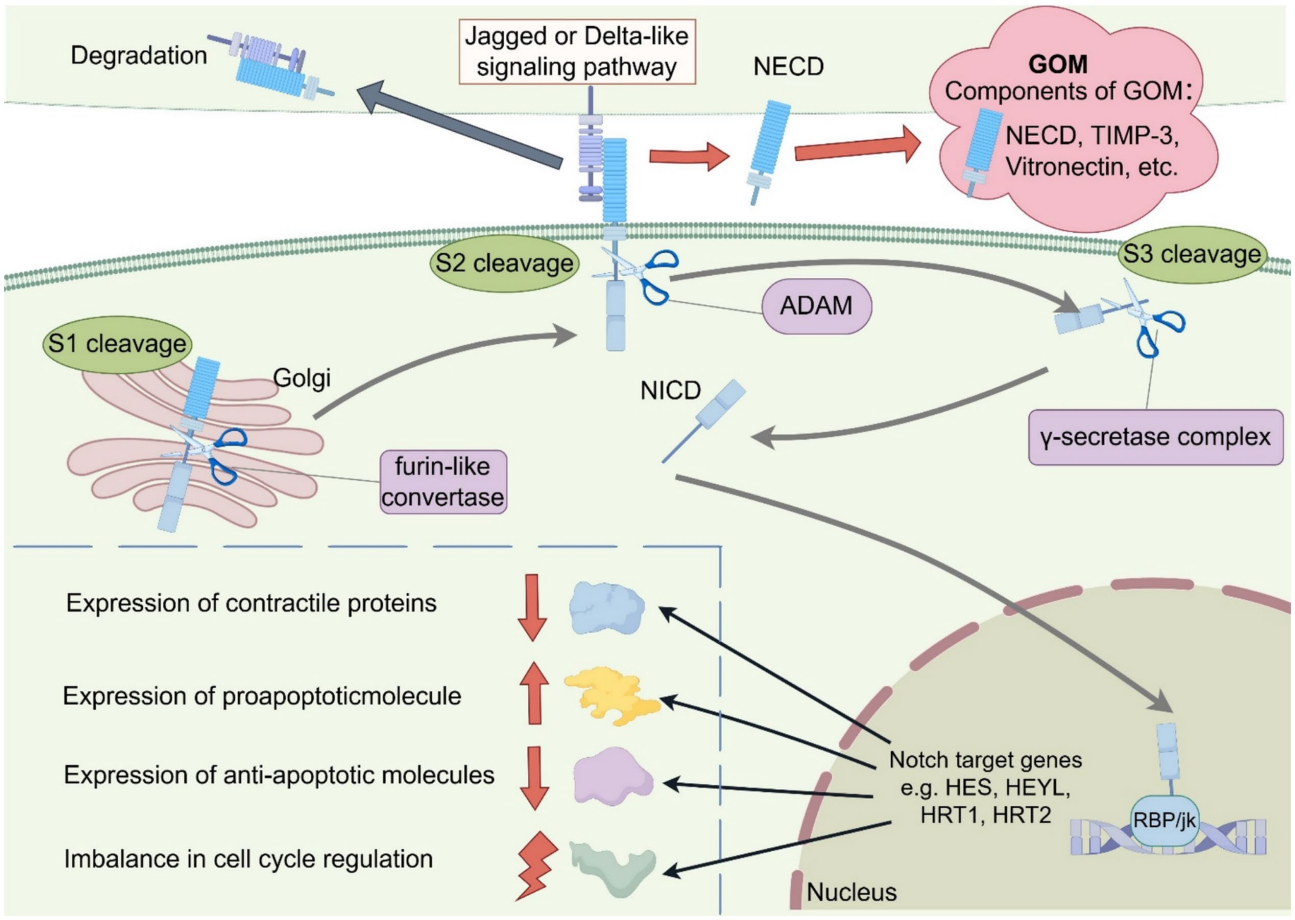
Schematic representation of the two key Pathogenesis of CADASIL. The schematic diagrams were designed by FigDraw (www.figdraw.com). The two main hypotheses being proposed are aberrant *NOTCH3* aggregation and aberrant *NOTCH3* signaling: (1) Mutations in the *NOTCH3* gene in CADASIL can lead to an increase or deletion in the number of 1 cysteine residues in EGFr. This unpaired cysteine residue disrupts normal disulfide bond formation, leading to misfolding of EGFr and increased *NOTCH3* multimerization, ultimately leading to the formation of GOM. In addition, proteins such as TIMP-3 and Vitronectin are involved in GOM formation. (2) In the NOTCH signaling pathway, the NOTCH receptor undergoes S1 cleavage by furin-like convertase in the Golgi apparatus and then transfers to the cytoplasmic membrane where it binds to Jagged or Delta-like ligands. When the NOTCH receptor binds to the ligand, it is cleaved by ADAM for S2 cleavage. After S2 cleavage the transmembrane structural domain and NICD enter the cytoplasm to participate in S3 cleavage induced by the *γ*-secretase complex, generating active NICD. NICD forms a complex with the transcription factor RBP/Jk, leading to the up-regulation of NOTCH target gene expression (HES1, HEYL, HRT1, HRT2, etc.), possibly by influencing the expression of contractile proteins, apoptotic factors and cell cycle proteins to regulate cell growth and apoptosis. GOM, granular osmiophilic material; NECD, NOTCH extracellular domain; NICD, NOTCH intracellular domain; ADAM, a disintegrin and metalloproteinase.

There is no consensus on the pathogenesis of CADASIL. Two key pathophysiological mechanisms are so far thought to contribute to the emergence and progression of CADASIL: aberrant *NOTCH3* aggregation and aberrant *NOTCH3* signaling (Figure 1). The typical pathologic feature of CADASIL is the presence of GOM deposits within or adjacent to the basement membrane of VSMC or pericytes by electron microscopy. GOM may disrupt VSMC function by inhibiting *NOTCH3*-regulated smooth muscle transcripts (24). Although the exact composition of GOM remains largely unknown, *in vivo* and *in vitro* studies suggest that *Notch3*^ECD^ is a major component of GOM. Meanwhile, extracellular matrix proteins such as Metalloproteinase inhibitor 3 (TIMP3) and Vitronectin (VTN) co-localized with *Notch3*ECD, which may jointly contribute to the formation of GOM deposits (25).

However, converging evidence suggests aberrant *NOTCH3* signaling as another potential pathogenic mechanism. The NOTCH signaling process is complex. The Notch receptor is first produced in the endoplasmic reticulum, and then undergoes its first cleavage in the Golgi apparatus by furin-like convertase. After cleavage it is transferred to the cytoplasmic membrane to bind to the five corresponding ligands (Jagged 1 and 2 and Delta-like 1, 3, and 4). When the Notch receptor binds to the ligand, specific sites within the negative regulatory region are cleaved by a disintegrin and metalloproteinase domain (ADAM)-type metalloproteinase, which is the second hydrolysis of the notch signaling pathway and a key step in its activation (26). The ECD is removed and taken up and degraded by the ligand-expressing cells, and the remaining transmembrane structural domains and the NICD participate in the third cleavage induced by the *γ*-secretase complex, generating active NICD. NICD forms a complex with the transcription factor RBP/Jk, leading to the upregulation of NOTCH target gene expression (HES1, HEYL, HRT1, HRT2, etc.), which may promote cell growth and regulate apoptosis (27). *NOTCH3* is expressed almost exclusively in vascular smooth muscle cells (VSMC). Aberrant *NOTCH3* signaling may affect VSMC differentiation, maturation, growth, and apoptosis by influencing the expression of contractile proteins, the expression of apoptotic factors (including pro- and anti-apoptotic molecules), and the expression of cell cycle proteins (28). A study by Celine et al. concluded that the p. Arg169Cys heterozygous missense mutation that causes CADASIL leads to *NOTCH3* hyperactivation (29). There is also evidence that *NOTCH3* mutations in EGFr10 to 11 that cause cysteine alterations not only cause GOM deposition, but also lead to defective *NOTCH3* receptor function, such as p. Cys455Arg located in EGFr10. A report from Colombia found that individuals with the p. Cys455Arg mutation exhibited ligand-induced *NOTCH3* signaling was severely reduced and an early-onset CADASIL phenotype (including early-onset stroke and extensive MRI abnormalities), which may be related to the attenuation of Jagged-1-induced *NOTCH3* receptor signaling through RBP-Jk by the p. Cys455Arg mutation (30). These studies suggest that while many mutations do not affect *NOTCH3* signaling, certain mutations appear to have varying effects on *NOTCH3* signaling activity, causing overactivity or attenuation of the signaling pathway. These diverse findings raise the possibility that different *NOTCH3* mutation types and locations can have different effects on *NOTCH3* signaling and can promote vascular abnormalities through different mechanisms (eg, loss of mural cells, etc). In models with *NOTCH3* mutations, either an increase or decrease in *NOTCH3* signaling may be interpreted in the context of a signaling threshold model (or goldilocks) (31). However, of all the current studies on the mechanisms of CADASIL, only a small number have systematically elucidated the effects of *NOTCH3* mutations on the *NOTCH3* signaling pathway. Therefore, in addition to GOM deposition, we need more studies to elucidate the role of *NOTCH3* signaling in pathogenesis.

## 4 Genotype–phenotype correlations in CADASIL

The core clinical manifestations of CADASIL include early and recurrent cerebral ischemic events (transient ischemic attack and ischemic stroke) and progressive vascular cognitive impairment. Other clinical manifestations include migraine with aura, mood disorders, gait disturbances, intracerebral hemorrhage, motor abnormalities (e.g., Parkinson’s disease), and seizures. Typical Brain magnetic resonance imagin (MRI) abnormalities are progressive symmetrical white matter hyperintensities (WMHs) involving the anterior temporal lobe, the external capsule, and the superior frontal gyrus, lacunar infarcts, cerebral microbleeds (CMBs), and cerebral atrophy (32). The diversity of mutations in the *NOTCH3* gene and clinical heterogeneity of the patients have revealed significant and complex genotype–phenotype correlations: mutations in *NOTCH3* mutations located in different EGFr regions lead to different severity of clinical phenotypes, the effect of some specific *NOTCH3* gene mutation types on clinical phenotypes as well as the fact that cysteine-conserving *NOTCH3* mutation types and classically altered cysteine number *NOTCH3* mutation types exhibit similar, but not identical, clinical phenotypes.

### 4.1 Effect of different mutation regions on disease phenotypes

To date, there have been many genotype–phenotype correlations on *NOTCH3* mutations caused by typical cysteine alterations. Several studies have been conducted to analyze the genotype–phenotype correlations from several common specific mutant types (Table 2).

**Table 2:** Comparison of clinical phenotypes of 5 common *NOTCH3* mutations (p. Arg141Cys, p. Arg182Cys, p. Arg75Pro, p. Arg544Cys, p. Pro167Ser).

	EGFr	Clinical manifestation								WMHs	Ref.
		Incidence of stroke/TIA	Onset of headaches	Onset of Cognitive impairment or dementia	Onset of mood disorders	Onset of epilepsy	Onset of gait disorders	Onset of abnormal behavior after headache	Asymptomaticity		
p. Arg141Cys	3	High	Common	Common	Uncommon	Uncommon	Uncommon	No report	No report	Common	(33)
p. Arg182Cys	4	Low	Common	Common	Common	Common	Uncommon	Common	Common	Common	(33)
p. Arg75Pro	1	Low (A Korean study concluded that it was high)	Uncommon	Common	Common	Uncommon		No report	Common	Uncommon	(33, 98, 99)
p. Arg544Cys	13–14	High (but late age of onset)	Uncommon	Common (not reported in a study in China)	Uncommon	Uncommon	Uncommon	No report	No report	Uncommon	(81, 82, 100, 101)
p. Pro167Ser	4	High	Common	Common	No report	No report	Uncommon	No report	No report	Common	(99, 102–104)

Rutten et al. found from a large-scale genetic variation database study that the frequency of EGFr cysteine-altered *NOTCH3* mutations in the general population was 3. 4/ 1,000, a frequency 100-fold higher than the currently estimated prevalence of CADASIL. Those individuals, in whom the mutation accumulates predominantly in EGFr 7–34, have an increased lifetime risk of developing stroke and vascular dementia, albeit with a later onset than in classic CADASIL patients. In contrast, in patients with CADASIL, typical cysteine-altering mutations are predominantly located in EGFr 1–6 (6). This led us to realize that the spectrum of diseases with *NOTCH3* mutations could be very broad, including CADASIL. subsequently, in a further study, Rutten et al. demonstrated that in patients diagnosed with CADASIL, mutations in EGFr 1–6 predispose to the classical, more severe CADASIL phenotype, whereas pathogenic variants in EGFr 7–34 more frequently lead to a milder phenotype. Specifically, patients with mutations in EGFr 1–6 had an earlier age of stroke onset by 12 years, shorter survival, and increased white matter high-signal volume compared with patients with mutations in EGFr 7–34, which is consistent with a previous study in Japan (33). Hack et al. reported a novel three-tiered risk classification for the *NOTCH3* variants that included not only EGFr structural domains 1–6 but also EGFr structural domains 8, 11, and 26 in high risk, as they found that patients with variants in EGFr structural domains 8, 11, and 26 had similar imaging burden and clinical presentation as patients with variants in EGFr structural domains 1–6 (34). A recent large cohort study not only confirmed the finding that pathogenic variants in EGFr 7–34 cause a milder clinical phenotype than pathogenic variants in EGFr 1–6, but further found that the severity of stroke was similar in EGFr 1–9 and 18–34, and that patients with variants in EGFr 10–17 had a lower risk of stroke than those with EGFr 3–4 (35). Similar to classical CADASIL, the location of mutations in the *NOTCH3* retention cysteine gene likewise influences the severity of the disease: exons 1–6 mutations present a more severe clinical manifestation compared to retention cysteine mutations in exons 7–34. This further explains the fact that the cysteine-sparing mutations that have been diagnostic of CADASIL to date are more frequently detected in exon 3: since these patients have more severe clinical manifestations and disease progression, they are more likely to be detected and diagnosed (8, 9).

The current study allows us to confirm location-dependent genotype–phenotype correlations in CADASIL, and that the location of *NOTCH3* pathogenicity variants is a key determinant of disease severity in CADASIL, above cardiovascular risk factors and gender, which is an important guideline for the development of a predictive model for *NOTCH3* to help us to identify these patients at risk for early onset, severe disease. We need to further explore the molecular mechanisms associated with the location of pathogenic variants of *NOTCH3*.

### 4.2 Effect of *NOTCH3* cysteine-sparing mutations on disease phenotypes

There are many reports of *NOTCH3* cysteine-sparing mutations with CADASIL-like clinical manifestations. Although the clinical presentation and imaging of patients with *NOTCH3* cysteine-sparing mutations are similar to those of classical CADASIL, they are not identical. Cysteine-sparing mutations have a later onset of symptoms than cysteine-associated mutations: the mean age of patients with CADASIL presenting with neurological signs or symptoms is 45–51. 3 year, particularly in men at approximately 50 years and in women at approximately 53 years (36), whereas carriers of *NOTCH3* cysteine-sparing mutations have a mean age of 53. 64 years. The most common clinical manifestations in patients with *NOTCH3* cysteine-sparing mutations are gait disturbance, cognitive impairment and stroke. The most common cerebral radiologic manifestations were lacunar cerebral infarction and cerebral microhemorrhage. Compared with patients with classical CADASIL, patients with *NOTCH3*-preserved cysteine mutations had a significantly higher frequency of cognitive impairment and cerebral microhemorrhages, whereas anterior temporal pole and external capsule white matter high signal was rarely observed. The relatively milder white matter involvement in two specific locations, the anterior temporal pole and the external capsule, may be an imaging feature of *NOTCH3*-preserved galactorrhea mutations (9). We speculate that this may be because the unique convoluted structure and vascularization of the temporal pole is particularly susceptible to myelin depletion or edema, which may result from aggregation of *NOTCH3*^ECD^ and microvascular changes (37). In patients with preserved cysteine mutations, these mutations are less involved in GOM deposition and have less effect on interstitial fluid drainage and white matter thinning. This may partly explain the different effects observed for different mutation types in the CADASIL phenotype. However, we need to standardize MRI protocols to ensure consistent detection and characterization of white matter lesions and vascular abnormalities across different patient populations. p. Leu1518Met is a variant located outside of the EGFr region that does not involve a change in the number of cysteines, and Park et al. have reported this variant in a GOM-positive patient with CADASIL (38). Schmidt et al. have linked the p. Leu1518Met variant to be associated with severe white matter lesions (39). p. Leu1518Met was detected in a 36-year-old female patient with severe neurological symptoms and a 47-year-old male patient, both of whom showed extensive white matter involvement on neuroimaging, suggesting that the p. Leu1518Met variant is associated with severe white matter lesions (40). In addition to this, patients with a homozygous *NOTCH3* mutation also seem to exhibit a clinical phenotype belonging to the classical clinical spectrum of CADASIL, whereas some reports have emphasized an increased disease severity in homozygous carriers, the exact mechanism of which is still unclear (41).

In summary, the location and type of mutation are major determinants of the clinical phenotype, and further studies are needed to clarify the relationship between CADASIL genotype and phenotype and the underlying mechanisms. However, the manifestation and severity of the disease cannot be explained solely by genotype–phenotype, but may also vary among different races and family lines with the same mutation (42), or even among identical twins (43). This may be related to modifier genes, environmental influences, or other concomitant risk factors that together determine the clinical heterogeneity of the disease.

## 5 Clinical polymorphisms in CADASIL

With the development of diagnostic and experimental techniques, we have identified *NOTCH3* mutations in more clinical studies, including anomalies of the large intracranial arteries, coronary artery disease and abnormalities in lipid and glucose metabolism. These clinical alterations may be related to the molecular mechanisms of *NOTCH3* signaling. Notch signaling is evolutionarily conserved, and the *NOTCH3* gene encodes the single-channel transmembrane receptor *NOTCH3*. Clinical manifestations of large intracranial arteries and extracranial have led to a reexamination of the CADASIL perspective and there is an urgent need for us to use new terminology to summarize this clinical polymorphism of *NOTCH3*.

### 5.1 Relationship between *NOTCH3* mutations and intracranial large arteries

In the past, it was thought that *NOTCH3* mutations caused pure cerebral small-vessel disease-classical CADASIL. However, several studies have shown that *NOTCH3* mutations may be present in a variety of intracranial large-artery anomalies causing infarcts associated with large-artery disease, with most of the patients having altered cysteine (44). Eun J (45) and Hyun (44) both confirmed in their clinical studies that intracranial large artery stenosis may not be an incidental phenomenon unrelated to mutations in the *NOTCH3* gene. In a study from China, 28 of 37 CADASIL patients (75. 7%) had intracranial large artery anomalies. Usually WMH distribution is symmetrical in CADASIL, but this study suggested that intracranial large artery disease in CADASIL patients may cause an asymmetrical WMH pattern, which may be due to large artery anomalies altering hemodynamics and exacerbating the WMH in the corresponding region. Therefore, this characteristic imaging manifestation could be an important marker of intracranial large artery stenosis in patients with *NOTCH3* (46). Hyun et al. suggested that intracranial large artery stenosis in patients with CADASIL may preferentially involve relatively small intracranial arteries because in these patients, the arterial stenosis is usually located in the anterior cerebral artery, the anterior inferior cerebellar artery, or the middle cerebral artery distal to the smaller vessels M1 and M2 (44). This clinical evidence suggests that *NOTCH3* mutations cause more than cerebral small-vessel disease and have an important association with intracranial large-artery stenosis, although most cases have been described only in East Asia. One autopsy showed GOM deposition in the aorta, carotid and renal arteries of a patient with CADASIL (47). An autopsy of a patient with CADASIL in Japan showed arterial stenosis in the basilar, internal carotid, anterior cerebral, middle cerebral, and posterior cerebral arteries, which exhibited atherosclerotic changes in the absence of any risk factors (48). Therefore, we hypothesized that stenosis of large intracranial arteries may be related to atherosclerosis, and that endothelial cell damage associated with GOM deposition accelerates the progression of atherosclerosis. In addition to stenosis of the great arteries, congenital anomalies of the intracranial great arteries in CADASIL patients have also been reported, including basilar artery fenestration, vertebral artery (VA) hypoplasia or agenesis, anterior cerebral artery (ACA) shares common trunk, and fetal posterior cerebral artery (FPCA). However, whether these congenital abnormalities of the cerebral vasculature share a common pathogenesis with the stenosis that develops later in life is unclear, and whether these changes are part of the *NOTCH3* mutant phenotype has not been further investigated (46).

The mechanism by which *NOTCH3* mutations cause atherosclerosis in large arteries has been less well studied. Atherosclerosis is a chronic inflammatory disease caused by the interaction of lipids, macrophages/foam cells and arterial wall cells (49). Chronic inflammation is central to the pathology of atherosclerotic vascular disease and metabolic disorders. In particular, macrophages are involved in the expanded cascade of reactions and play a key role in maintaining the inflammatory response in atherosclerotic plaques, promoting their structural instability, and thrombus formation (50). There are multiple *NOTCH3* receptors and ligands expressed in macrophages, among which DII4 and *NOTCH3* are co-localized in macrophages within atherosclerotic plaques (51). Fung et al. revealed the role of the DII4-NOTCH pathway in the inflammatory response characterized by macrophage activation: stimulation of pro-inflammatory factors such as LPS and IL-1β increased the expression of DII4 in macrophage expression, triggered hydrolysis and activation of NOTCH proteins, and induced pro-inflammatory molecules and pathways, such as inducible nitric oxide synthase (iNOS), pentraxin 3 (PTX3), mitogen-activated protein kinase (MAPK), Akt, and NF-κB. Notably, DII4-triggered NOTCH signaling increased the expression of DII4 itself, which further demonstrates that the DII4-NOTCH signaling pathway mediates the inflammatory response by accelerating is a positive feedback loop of cell activation leading to atherosclerotic plaque loading, progression and thrombus formation (52). Consistently, blockade of Dll4-Notch signaling using a neutralizing anti-Dll4 antibody attenuated the development of atherosclerosis in LDL receptor-deficient mice (53). VSMC influence atherosclerotic plaque formation in several ways and may determine susceptibility to plaque rupture (54, 55). Among these, trans-differentiation of VSMCs, or their transition from a contractile/quiescent state to a secretory/inflammatory/migratory state plays an important role during atherosclerosis (56). Although the relationship between VSMC and NOTCH signaling during atherosclerosis is unclear, it has been found that atherosclerotic plaques in human carotid and femoral arteries usually have a lot of *NOTCH3* proteins, and it is possible that Jagged1 stimulates the NOTCH signaling pathway in VSMCs, which causes pathological changes (57). Adenylate cyclase type 8 (AC8) is known to be involved in trans-differentiation of VSMC. A study by Zela et al. indicated that Jagged1-triggered inhibition of the *NOTCH3*-HRT3 and/or HRT1 pathways in the context of pathological vascular remodeling enhanced IL1β-mediated up-regulation of AC8 in trans-differentiated VSMC and amplified its deleterious effect on trans-differentiated VSMC differentiation (58). A recent study by Dave et al. linked elastin deficiency to NOTCH signaling (59): although elastin deficiency does not directly contribute to atherosclerosis, degradation of elastin may allow lipid infiltration into atherosclerotic plaques; elastin deficiency in VSMC resulted in reduced levels of DNA methyltransferase 1 (DNMT1) and DNA methylation (an epigenetic marker that drives gene silencing), which induces the expression of key NOTCH pathway genes Jagged1, *γ*-secretase catalytic subunits PSEN1 and PSEN2 and triggers *NOTCH3* activation. That is, upregulation of the *NOTCH3* pathway causes accumulation of pathological VSMC during elastin deficiency (59). Therefore, we suggest that lesions of large intracranial arteries may be one of the clinical phenotypes of *NOTCH3* mutant disease. The study of the molecular biological mechanism of *NOTCH3* mutation-induced lesions in large vessels led us to hypothesize that *NOTCH3* mutation is a general vascular lesion affecting all types of intracranial vessels but manifesting predominantly in small vessels rather than a lesion unique to small vessels. We need to further investigate the mechanism and validate it pathologically in stenotic vessels.

### 5.2 Association of *NOTCH3* mutations with coronary artery disease

To the best of our knowledge, although the vascular lesions in classical CADASIL predominantly involve the intracranium, vascular changes in CADASIL occur along the arterial tree, including the heart (47). Embryological effects of the *NOTCH3* receptor pathway responsible for the development of the cardiovascular system support this theory (60). Consistent with the findings of CADASIL intracranial vasculature, we confirmed abnormalities of the coronary microvascular system in myocardial histopathologic examination and found typical CADASIL changes such as GOM deposition, cellular degeneration, and basement membrane thickening (61). In recent years an increasing number of patients with CADASIL present with ischemic coronary artery disease (CAD), and there have been many reports of an association between CADASIL and CAD, albeit with limited evidence. Argirò et al. included 17 patients with CADASIL confirming a blunted coronary flow reserve due to dysfunction of the coronary microcirculation with diffuse hypoperfusion areas (62). In a case report of a patient with CADASIL presenting with angina pectoris without obstructive coronary artery disease, coronary CT angiography demonstrated diffuse vascular irregularities, vascular thickening, and mixed noncalcified/calcified coronary lesions with moderate stenosis (63). Consistently, Rubin et al. performed coronary angiography in patients with CADASIL and found diffuse and irregular stenosis of the involved coronary arteries (64). Thus, these clinical findings suggest that *NOTCH3* mutations may have caused coronary microvascular dysfunction and diffuse coronary stenosis rather than coronary atherosclerosis. Also, the more aggressive and accelerated CAD in CADASIL patients may be related to microvascular dysfunction, allowing for a younger age of onset and a broader disease burden (65). *NOTCH3* mutations impair the damaged heart through coronary microvascular dysfunction, loss of pericytes, and impaired microvascular maturation recovery, and the main causes of coronary microvascular dysfunction are fibrovascular wall thickening, impaired vascular reactivity, and increased myogenic tone (66).

Several studies have been conducted on the molecular mechanisms of how *NOTCH3* mutations cause CAD. Mutations in components of the NOTCH pathway lead to structural abnormalities and dysfunction in the heart. In particular, activation of NOTCH exerts a protective effect against myocardial infarction, cardiac hypertrophy, and alcoholic cardiomyopathy. Binding of *NOTCH3* receptors on human coronary smooth muscle cells and Jagged1 ligands on human coronary endothelial cells contributes to increased expression of smooth muscle-*α*-actinin and calmodulin (67). A recent study demonstrated that the *NOTCH3*-RBPJk signaling pathway maintains the integrity of the coronary vascular system by regulating the vascular endothelial growth factor A (VEGFA)/vascular endothelial growth factor receptor 2 (VEGFR2)/DII4 pathway. Thus, the absence of the *NOTCH3*-RBPJk signaling pathway in VSMC leads to heart failure during pressure overload, and alterations in the cardiac vasculature are a direct causative factor (68). Oncostatin M (OSM) is known to be an inflammatory cytokine, and inhibition of OSM signaling inhibits cardiomyocyte remodeling, leading to deterioration of cardiac function after myocardial infarction. A study showed that the mechanism of OSM on cardiac ischemia/reperfusion injury is partially mediated by the *NOTCH3*/Akt pathway. Thus, a novel role of *NOTCH3*/Akt signaling contributes to OSM-induced protection against cardiac ischemia/reperfusion injury (69).

### 5.3 Association of *NOTCH3* mutations with metabolic abnormalities such as lipids and blood glucose

NOTCH signaling is considered a key player in metabolism (70), and activation of the *NOTCH3* gene may lead to hyperlipidemia. Gong et al. reported 1 family with CADASIL with elevated lipoprotein levels that caused a higher risk of disease (71). Apolipoprotein D (APOD) is known to be a secreted glycoprotein that regulates smooth muscle cells (72, 73). It is associated with lipid metabolism, particularly in plasma mainly associated with High-density lipoprotein HDL, and is abundantly present in atherosclerotic lesions (74). Several studies have demonstrated a correlation between APOD expression and healthy lipid profiles (75). It was shown that cell contact-dependent Notch signaling contributes to the regulation of APOD in mural cells (76). Further experiments revealed that *NOTCH3* contributes to the down-regulation of APOD, possibly through the activation of the downstream NOTCH signaling pathway by JAGGED-1 ligand on endothelial cells, which increases the regulation of APOD in wall cells by endothelial cells (77). Therefore, we suggest that mutations in the *NOTCH3* gene may cause impairment of the corresponding signaling pathway and increased APOD expression, leading to severe dyslipidemia. Wimmer et al. identified the Dll4-*NOTCH3* pathway as a key driver of diabetic vasculopathy in the human vasculature. They constructed vascular organoid models and placed them in a high-glucose environment and observed by electron microscopy a marked increase in collagen IV, as well as fibronectin, laminin, perlecan, and the pro-inflammatory cytokines TNFα and IL6. Mainly pericytes, but also endothelial and MSC-like cells, upregulated ECM synthesis. And we also observed substantial thickening and splitting of the basement membrane layer without changes in vessel diameter. Basement membrane thickening was reduced after blocking *NOTCH3* protein and its ligand DLL4, suggesting that the DLL4-*NOTCH3* pathway plays an important role in diabetic vasculopathy (78). We speculate that *NOTCH3* gene mutations may interfere with the Dll4-*NOTCH3* pathway thereby causing blood glucose abnormalities, which suggests that we investigate the association between the CADASIL clinical phenotype and diabetes through more clinical series. However, there are limited studies on the relationship between *NOTCH3* mutations and metabolisms such as lipids and glucose, and we need further studies on pathogenesis to clarify the relationship.

In addition to this, *NOTCH3* has many associations with human diseases through mutations, altered expression, or dysregulation of its activity or turnover. Although the main function of *NOTCH3* is to regulate angiogenesis, dysregulation of *NOTCH3* activity may play an important role in tumorigenesis and tumor maintenance. The apparent alteration (and in many cases overactivation) of *NOTCH3* signaling activity causes a number of changes, including controlling the tumor-initiating cell phenotype, regulating known upstream or downstream tumor-associated signaling factors, facilitating angiogenesis or tumor invasion, regulating the cell cycle, etc. Recent studies have found that *NOTCH3* expression is found in most T-cell acute lymphoblastic leukemias, possibly through the activation of *Notch3* signaling by the expression of Dll in endothelial cells thereby promoting the proliferation of T-cell acute lymphoblastic leukemia cells (79). Furthermore, it has been demonstrated that aging is associated with a significant progressive decline in *NOTCH3* signaling. This age-related lack of *NOTCH3* triggers a decline in vascular function, which subsequently affects lymphatic flow and ultimately leads to degenerative changes (80). Thus, the complex and highly variable functions of *NOTCH3* factors have led to diverse associations in human development and disease, and the pathogenesis of these *NOTCH3*-related diseases almost always involves alterations in the *NOTCH3* signaling pathway. However, whether these diseases share the same mechanisms as CADASIL has not been systematically elucidated. Perhaps, further studies of the mechanisms and a more complete understanding of NOTCH signaling will help to generalize the clinical heterogeneity of NOTCH mutations. At the same time, this will underpin the development of means to more specifically target its aberrant activity in different disease contexts.

To date, there have been no case reports of CADASIL patients with concomitant intracranial large artery and coronary artery stenosis. However, previous studies have found typical CADASIL pathologic manifestations such as deposition of GOM in both intracranial large arteries and coronary arteries. Meanwhile, activation or inhibition of the NOTCH signaling pathway caused by *NOTCH3* mutations also leads to pathological changes such as VSMCs. In conclusion, *NOTCH3* mutations may lead to intracranial aortic anomalies, coronary artery disease, and abnormalities in lipid and glucose metabolism through different mechanisms. *NOTCH3* mutations may no longer be simply a disease of the small blood vessels of the brain-typical of CADASIL-but a new spectrum of diseases that share the same pathogenesis as *NOTCH3* mutations. However, there is evidence that traditional risk factors such as hypertension, diabetes mellitus, or smoking also contribute to the more severe clinical phenotype of CADASIL. It is possible that the observed cases of intracranial large artery stenosis or coronary artery disease reflect traditional vascular risk factors and diseases (e.g., higher prevalence of intracranial atheromatous plaques in Southeast Asia) rather than representing a true characterization of the pathology associated with *NOTCH3* mutations. Therefore, more robust clinical studies and mechanistic investigations are needed to demonstrate that clinical polymorphisms do indeed characterize genetically determined diseases.

## 6 Conclusion

In conclusion, this study synthesizes the CADASIL mutational landscape and systematically delineates genotype–phenotype correlations with their underlying pathophysiological mechanisms. The correlation between CADASIL genotypes and phenotypes depends mainly on the *NOTCH3* mutation location and mutation type. Environmental factors, epigenetic regulation and multigene interactions also influence the correlation to some extent. Despite great progress, the exact mechanisms have not been clarified. Continued future research on CADASIL genotype–phenotype correlation is essential to reveal its heterogeneity, identify therapeutic targets, achieve early diagnosis and mitigate disease progression. Moreover, we further demonstrated that *NOTCH3* mutations cause anomalies of the large intracranial arteries, coronary artery disease, and metabolic abnormalities through a pathogenesis similar to that of classical CADASIL. Unfortunately, this association has not been confirmed by large clinical series and potential mechanistic studies. Still, these findings suggest a possible need to revisit the concept of CADASIL (characterized by cerebral small-vessel disease caused by *NOTCH3* mutations) as a disease. We attempt the establishment of a new term- *NOTCH3* mutaion spectrum diseases -to explain this novel disease entity that differs from traditional CADASIL. It refers to *NOTCH3* mutation-associated diseases that contain multiple heterogeneous presentations with similar pathogenesis. The application of this terminology is expected to provide a more comprehensive overview of the complexity and heterogeneity of *NOTCH3* mutation diseases and support the development of precision medicine approaches. Future studies will explore the association of *NOTCH3* mutations with intracranial large arteries, coronary artery disease, and metabolic abnormalities and their associated pathogenesis based on a larger sample.
